# Bio-Inspired Algorithms for Efficient Clustering and Routing in Flying Ad Hoc Networks

**DOI:** 10.3390/s25010072

**Published:** 2024-12-26

**Authors:** Juhi Agrawal, Muhammad Yeasir Arafat

**Affiliations:** 1 School of Computer Science, University of Petroleum & Energy Studies, Prem Nagar, Dehradun 248007, India; 2 IT Research Institute, Chosun University, Gwangju 61452, Republic of Korea

**Keywords:** aquila optimizer, bio-inspired algorithm, clustering, FANETs, mountain gazelle optimizer, routing, unmanned aerial vehicles, UAV networks

## Abstract

The high mobility and dynamic nature of unmanned aerial vehicles (UAVs) pose significant challenges to clustering and routing in flying ad hoc networks (FANETs). Traditional methods often fail to achieve stable networks with efficient resource utilization and low latency. To address these issues, we propose a hybrid bio-inspired algorithm, HMAO, combining the mountain gazelle optimizer (MGO) and the aquila optimizer (AO). HMAO improves cluster stability and enhances data delivery reliability in FANETs. The algorithm uses MGO for efficient cluster head (CH) selection, considering UAV energy levels, mobility patterns, intra-cluster distance, and one-hop neighbor density, thereby reducing re-clustering frequency and ensuring coordinated operations. For cluster maintenance, a congestion-based approach redistributes UAVs in overloaded or imbalanced clusters. The AO-based routing algorithm ensures reliable data transmission from CHs to the base station by leveraging predictive mobility data, load balancing, fault tolerance, and global insights from ferry nodes. According to the simulations conducted on the network simulator (NS-3.35), the HMAO technique exhibits improved cluster stability, packet delivery ratio, low delay, overhead, and reduced energy consumption compared to the existing methods.

## 1. Introduction

Flying ad hoc networks (FANETs) are critical in modern applications that require high flexibility, fast deployment, and real-time communication in dynamic environments, such as disaster response, environmental surveillance, search and rescue, agriculture, and tactical military operations [1,2,3,4,5,6]. Within this environment, unmanned aerial vehicles (UAVs) establish decentralized networks to share information between nodes and provide end-to-end communication over large and often inaccessible spaces [7,8,9]. However, the unique complexity and high mobility of UAV nodes with dynamic topologies pose challenges for FANETs [10]. FANETs face several difficulties, including frequent link failures, latency, and inefficient energy consumption [11,12,13]. Existing clustering and routing protocols are primarily based on a single optimization technique, such as geographical positioning or basic energy metrics [14]. Despite their ability to perform well in static or low-mobility networks, they are weak in high-mobility FANET environments [15,16]. Inefficient handling of frequent re-clustering or dynamic maintenance of routes creates problems with scalability and adaptability [17,18]. This causes more overhead, and as a result, diminished stability in the network [19]. Thus, an effective solution that allows for adaptability within a framework for multi-objective optimization to ensure cluster stabilization, promising predictability, and low latencies among routing features is required.

All the clustering and routing algorithms developed so far have been found to be able to support specific aspects of FANET performance, but fail in their application in high-mobility environments where frequent re-clustering and route maintenance cause increased latency and relatively reduced network stability [20]. Most existing deployed methods rely on a few metrics, such as geographical positioning or residual energy, which are not sufficient for dynamic adaptive FANETs. This inability reflects the need for a better, multi-objective-based solution that can handle both clustering and routing dynamically [21].

The exploding nature of FANETs comprising UAVs has posed tremendous challenges in providing reliable, scalable, and energy-efficient communication. The problems associated with networks in FANETs are inherent because of the mobility of UAVs, changing topologies, and the associated frequent link failures and overhead communication [22]. The traditional approach to clustering has been proven to be crucial for the purpose of network scalability and reduction in energy consumption, but fails to account for dynamic network conditions. It mainly works based on local information, such as the residual energy or proximity. Thus, it fails to consider the cluster size. Re-clustering must be carried out every time the cluster head (CH) moves out of range, there is a drop in energy levels, or imbalances in the cluster size. Such highly imbalanced clusters lead to a high node overload in densely populated regions, causing congestion in communication [23]. Existing protocols are not efficient in scenarios involving mobility-induced link failure and efficient data delivery under high-density or high-mobility conditions. Energy consumption is inefficient, packet loss is high, and networks lack fault tolerance, leading to unreliable performance. Therefore, the adaptation and robustness of an algorithm to optimize clustering and routing in FANETs with global network information becomes of prime importance [24,25].

To overcome these disadvantages, this paper proposes a novel hybrid bio-inspired algorithm based on the mountain gazelle optimizer (MGO) and aquila optimizer (AO), named HMAO, which combines local and global optimization techniques with the stringent requirements of FANETs. It uses the MGO algorithm for energy-efficient stable CH selection to ensure that the adaptation of the clustering process according to network conditions is still optimal in terms of energy usage. In addition, the AO algorithm applies mobility-predictive adaptive multi-hop routing to eliminate link failures and rerouting. The AO is a global search algorithm motivated by the hunting behavior of the aquila eagle, which covers large search spaces efficiently to find optimal solutions. It focuses on local optimization to stabilize clusters in dynamic networks, inspired by the agility of gazelles. HMAO using dual optimization has also been further improved by an innovative density-aware clustering mechanism that can be adjusted in real time to cluster size and density along with UAV mobility. This function is set to reduce the number of re-clustering events and, consequently, the routing overhead and stability of communication links, especially in highly dynamic scenarios with fast node movement.

### 1.1. Contribution of This Study

This paper introduces a novel solution, a hybrid bio-inspired algorithm called HMAO, which is designed to address the persistent problems stated above, improve cluster stability, and enable predictive routing within dynamic FANETs.

- In this study, an HMAO algorithm is proposed to optimize clustering and routing in FANETs by merging the best features of MGO for CH selection and AO for global path optimization.
- Local density-aware clustering is proposed to avoid overloading UAVs in dense regions. The proposed method ensures that cluster sizes are balanced and prevents congestion in high-traffic areas.
- A predictive mobility-based routing mechanism is proposed to predict UAV movement, and this mechanism will improve data delivery performance and reduce the packet loss rate in a highly dynamic environment.
- Proposed HMAO dynamically balances the data load across UAVs by monitoring queue lengths and packet processing rates and directing traffic towards the least-congested nodes. This prevents overwhelming, reduces delays, and improves network efficiency, especially in dense scenarios.
- The fault-tolerant secondary path routing strategy is designed with the ability to dynamically activate secondary paths on congestion occurrences for the reliable transmission of data using global information.

### 1.2. Organization of This Paper

The remainder of this paper is organized as follows. In Section 2, we provide a thorough review of related work, summarizing the existing gaps in FANET clustering and routing. Section 3 presents the system model along with the assumptions for the proposed HMAO algorithm. In Section 4, we describe the details of the proposed HMAO algorithm and its clustering and routing functionalities. Section 5 evaluates the proposed method through intensive simulation studies and compares it with several state-of-the-art algorithms. Finally, in Section 6, we conclude the study with key findings and future research directions.

## 2. Related Works

Related works highlight various approaches and advancements in clustering and routing techniques for FANETs. Although these previously proposed methods show significant improvements in energy efficiency, data delivery, and network stability, they lack scalability, adaptability to high mobility, and effective use of geographical information. A comparative analysis is thus provided to gain a clearer view of how our proposed HMAO algorithm fits the research.

Hosseinzadeh et al. [26] proposed energy-aware routing with virtual relay tunnels (EARVRT), which has the potential to control overheads for control messages using virtual relay tunnels for controlling updates better. Through this power-saving mechanism, redundant transmissions are avoided, thereby improving delivery quality and minimizing latency. Despite its applicability to medium-mobility FANETs, and that its performance is acceptable under controlled conditions, it lacks scalability because it is incompatible with larger networks and even highly dynamic environments such as UAVs. Hosseinzadeh et al. [27] proposed a local filtering-based energy-aware routing scheme (LFEAR), which controls routing overhead while maintaining stable paths. The filtering method reduces the number of requests for routes and the amount of energy consumed, and maintains a higher degree of adaptability in medium-density networks. Despite its effectiveness in enhancing route stability, this strategy has resulted in an increased overhead in dense FANETs. In [28], the authors proposed a K-means-based mobility-aware routing protocol (K-MORP), where they utilized the K-means algorithm to generate clusters that help in efficient and scalable routing in mobility-intensive FANETs. Clustering simplifies the routing overhead by managing UAVs in groups, thus helping to allocate resources and reduce the complexity of routing. However, at higher UAV speeds, the dynamic clustering stability becomes a problem. Therefore, K-MORP is effective in controlled or semi-structured environments. This protocol is very useful in cases with moderate movement but very limited in high-mobility FANETs.

In [29], the authors proposed a mobility-based weighted clustering scheme (MWCRSF) aimed at improving the routing efficiency in FANETs by optimizing weighted factors, such as node density and UAV mobility. Owing to its high computational complexity, the MWCRSF is not well suited to highly dynamic environments and has excellent scalability for large networks. Smith et al., [30] proposed a dynamic clustering for fault handling (DCFH) method that targets the reliability of FANETs by dynamically adapting clusters to handle faults and ensure data integrity during communication. This approach is fault-tolerant because the network continues to work even if individual UAVs have connectivity problems. Using whale optimization algorithms (WOA) for FANETs, the authors in [31] proposed an intelligent clustering scheme (ICW). ICW consists of centralized clustering that uses WOA to determine the best cluster centers within a network. Using the closeness ratio, ICW determines which UAV belongs to which cluster, so that each UAV belongs to the cluster with the best closeness ratio. The closeness ratio indicates the relative proximity of nodes within a network. Typically, it is calculated as the reciprocal of the sum of the shortest path distances between each node in a network. In [32], the authors proposed a utility function-based greedy perimeter stateless routing method called the UF-GPSR protocol for FANETs. This method optimizes greedy forwarding strategies based on multiple vital UAV parameters, including the residual energy ratio, distance degree, movement direction, link risk degree, and speed.

In recent years, bio-inspired algorithms have been used to solve the clustering problem in FANETs. Owing to their simplicity, effectiveness in solving complex optimization problems, and ability to avoid local minima, they have gained popularity. Most of these algorithms are based on animal behavior, evolutionary concepts, and physical phenomena. These schemes aim to solve clustering challenges, such as dynamic network topology, energy efficiency, and load balancing, by mimicking the adaptability, efficiency, and robustness of natural systems. Load balancing techniques are widely used in routing across various types of networks, including FANETs. In routing, load balancing refers to the practice of efficiently distributing network traffic across multiple paths or nodes to maximize network performance. In [33], the authors proposed a novel dynamic clustering mechanism called DCM utilizing a political optimizer. DCM addresses UAV load balancing to facilitate the efficient distribution of data packets within FANETs while ensuring good reliability and scalability. Using glowworm swarm optimization (GSO) to form and manage clusters, Khan et al. [34] proposed a self-organization-based clustering scheme (SOCS) for FANETs. Moth flame optimization (MFO) offers excellent coverage, with minimal energy consumption and a minimal number of CHs required for routing. The authors of [11] used the MFO algorithm for network building and node deployment in FANETs. A novel clustered routing model for FANETs was proposed in [35], utilizing a hybrid approach from a MGO with Jaya Algorithms, called HMGOC (MGO-JAYA). There are many advantages to using HMGOC, including improved load balancing, reduction in energy consumption, reduction in latency, and increase in network throughput. In [36], the authors proposed a bio-inspired clustering scheme called BICSF for FANETs that utilizes a hybrid mechanism consisting of GSO and krill herd (KH). Using the GSO algorithm, the proposed scheme uses energy-aware cluster formation and CH selection. In addition, in a behavioral study of KH, the authors proposed an efficient cluster-management algorithm.

In Table 1, we summarize all previously existing state-of-the-art methods and their objectives, strengths, and weaknesses. A range of methods has been discussed in the existing literature for routing efficiency, energy conservation, and stability within FANETs. However, these approaches are not easily scalable or adaptable to such a highly dynamic and mobile FANET environment. To address these limitations, this paper proposes the HMAO algorithm to integrate the advantages of these methods with regard to gaps in adaptability and scalability and offers a comprehensive solution specifically for the complex demands of FANETs.

## 3. Preliminaries

The purpose of this section is to provide preliminary knowledge regarding the proposed HMAO to facilitate its understanding. The first step is to discuss a motivational scenario, network model and assumptions, and energy model. Next, a framework for HMAO of FANETs is presented.

### 3.1. Motivation Scenario

**Figure 1:**
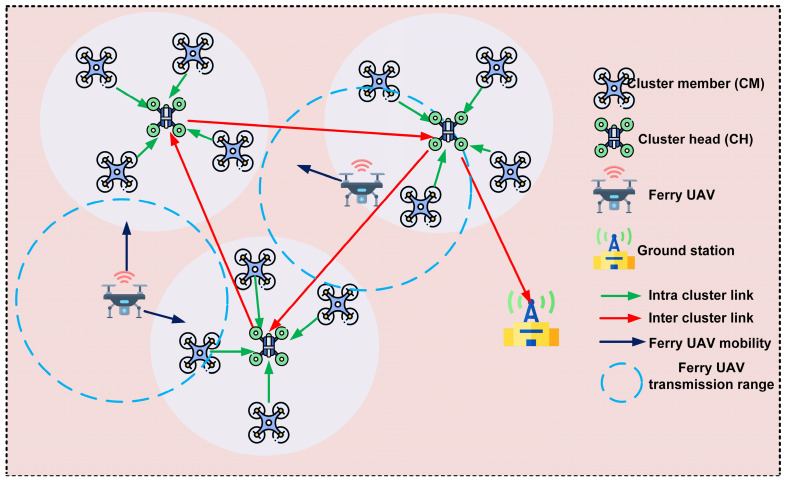
Demonstration of UAV clustering and routing in FANETs: Cluster members (CMs) are connected to their respective cluster heads (CHs) within each cluster, while CHs communicate with each other. Furthermore, ferry UAVs establish high-throughput connectivity between CHs and the ground station.

In this study, we adopted the typical network model shown in Figure 1. Figure 1 shows how UAVs in FANET are structured into different clusters. The CHs manage the clusters by authorizing various nodes to communicate with each other within the cluster and forward data between them. Furthermore, our approach includes ferry UAV nodes that gather and distribute global network data, such as the location and load of other UAVs. This allows decisions to be made adaptively, based on a network view. A predefined mobility path is followed by ferry UAVs within the network. Data gathered from ferry nodes contain a variety of network metrics, including UAV position and load information. Utilizing this information, routing decisions can be made more effectively.

### 3.2. Network Models and Assumptions

The network consists of N UAVs flying in three dimensions (3D) space, each represented by its position ($x_{i},y_{i},z_{i}$) and velocity (${Vx}_{i},{Vy}_{i},{Vz}_{i}$). A simplified collision technique [24] is used in the simulation to avoid collision. In this simplified collision technique, the UAV changes its altitude in the event of a potential collision. A UAV can reach speeds up to 30 m/s at its highest point. UAVs are equipped with wireless communication interfaces, global positioning systems (GPS), and inertial measurement units (IMU). GPS and IMU are provided for the positioning and motion sensing of UAVs. UAVs communicate with each other if they are within the communication range $R_{max}$. The distance between UAV i and UAV j is calculated as follows:

(1) $$d_{ij}=\sqrt{{\left(x_{i}-x_{j}\right)}^{2}+{\left(y_{i}-y_{j}\right)}^{2}+{\left(z_{i}-z_{j}\right)}^{2}}.$$

If $d_{ij}\leq R_{max}$, UAVs can communicate directly; otherwise, multi-hop communication is required. Every UAV is aware of its position, its neighbors’ position, and the position of its ground station. Every UAV is assigned a distinct identifier ($U_{ID}$) for unique identification within the network. UAVs move predictably over short time intervals, allowing future position estimation based on current velocity. Mobility stability is sufficient for selecting stable CHs and maintaining cluster consistency. Ferry nodes periodically collect and distribute global information such as network congestion and UAV positions to support dynamic clustering and routing decisions. Congestion may occur when UAVs are concentrated in a small area, or when a CH is overloaded with traffic.

### 3.3. Energy Consumption Model

Each UAV has an initial energy $E_{max}$ and residual energy $E_{i}(t)$ at time t, which depletes as the UAV transmits data, moves, and performs other tasks. The energy consumed during communication is calculated as follows

(2) $$E_{con}=E_{tx}+E_{rx},$$

where $E_{tx}$ and $E_{rx}$ represent the total energy a UAV expends during the transmission ($E_{tx}$) and reception ($E_{rx}$) of data packets, respectively. It is crucial to manage UAVs’ energy efficiency and overall network longevity of the UAVs. The total energy consumption of all UAVs in the network is defined as follows:

(3) $$E_{total}=\sum_{i=1}^{N}E_{i}(t).$$

The objective is to minimize $E_{total}$ while selecting UAVs with sufficient energy to serve as CH as follows:

(4) $$E_{i}\left(t\right)>E_{th}\;\forall_{i}\in CH,$$

where $E_{th}$ is the threshold energy level of the node i. The energy model in the HMAO framework calculates the total energy consumed in both single-path and multi-path routing, accounting for the transmission and reception costs based on distance and path selection. For single-path routing, the total energy consumption is defined as follows:

(5) $$E_{total\_single}=\sum_{k=1}^{n}E_{tx}\left(l,d_{k}\right)+E_{rx}(l),$$

where n is the number of hops, $E_{tx}\left(l,d_{k}\right)$ is the transmission energy for each hop k, and $E_{rx}(l)$ is the reception energy level. Energy consumption for multi-path is defined as follows

(6) $$E_{total\_multi}=\sum_{p=1}^{m}\sum_{k=1}^{np}E_{tx}\left(l,d_{k,p}\right)+E_{rx}(l),$$

where m is the number of paths, np is the number of hops in path p, and $d_{k,p}$ is the distance for each hop in each path.

### 3.4. HMAO Model

The proposed HMAO model aims to optimize both clustering and routing for FANETs with high dynamics. In FANETs, the frequencies of topology changes are far higher, UAV mobility is very high, and resources are scarce. Therefore, a model has been developed which incorporates two types of optimizations: MGO and AO for specific functions related to network operations. MGO is selected based on the strength of the local optimization for the selection of CHs in dynamic environments. Inspired by the adaptive strategies of mountain gazelles, an MGO can navigate variable conditions such as the selection of stable and energy-efficient CHs in an ever-changing network. Focusing on local metrics such as residual energy and mobility stability ensures that MGO selects the most suitable UAVs as the CH. This ensures maximum energy efficiency and stable intra-cluster communication by reducing the likelihood of re-clustering.

**Figure 2:**
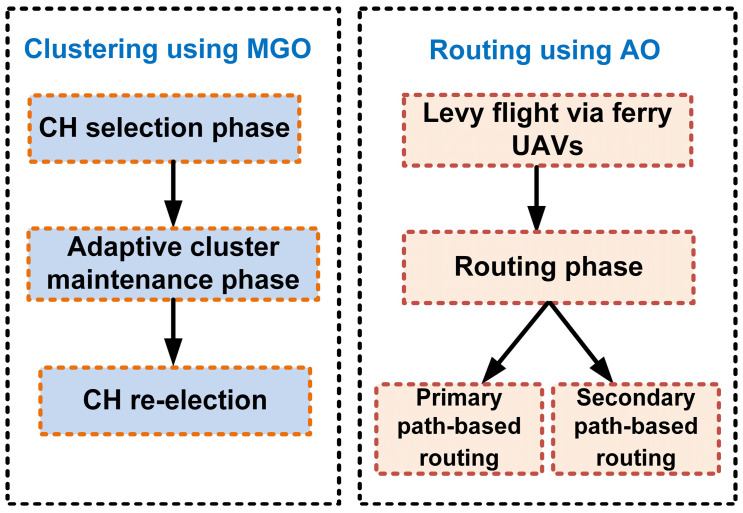
Block diagram of proposed system.

AO is incorporated because of its strong exploratory power and is used in global-path optimization. The aquila eagle hunting strategy inspires the AO to detect optimal routes across large areas of search and is highly applicable to multi-hop routing in FANET. AO utilizes global information gathered by ferry nodes, such as the UAV position and load. This enables data to be routed along paths that can minimize delays and avoid congestion. The HMAO algorithm combines local and global optimization strengths to enhance both the clustering and routing processes. For MGO, local optimization helps stabilize clusters, but with AO on global optimization, data may be effectively routed through the network. These dual-layer approaches can further stabilize a network with minimal energy usage and packet loss. It therefore endures high mobility along with the dynamic network changes observed in the FANET. The block diagram of HMAO is illustrated in Figure 2.

## 4. HMAO Algorithms

In this section, the HMAO-based clustering and routing for FANETs are discussed in detail. The comprehensive structure of HMAO ensures that the algorithm is robust and adaptable, thereby meeting the unique demands of FANETs while addressing the limitations of existing clustering and routing methods.

### 4.1. Clustering and CH Selection Phase

The MGO algorithm is employed to identify the optimal CHs to efficiently establish clusters in highly dynamic FANETs. This selection process, which is vital for cluster stability, leverages local optimization metrics such as energy levels, mobility stability, and connectivity to ensure that the chosen CHs support sustainable and low-overhead network operations. In the initial phase, UAVs are assigned to clusters and the most suitable CHs are selected. Each UAV is evaluated based on key metrics such as the energy level, mobility, and distance to other UAVs in the cluster.

The energy component of the fitness function is used to evaluate the residual energy of the UAV. A higher energy level is preferable because it ensures that the UAV can function longer as a CH without depleting its battery. The energy for UAV *i* is calculated as the normalized residual energy as follows

(7) $$E\left(i\right)=\frac{E_{residual,i}}{E_{max}},$$

where $E_{residual,i}$ is the remaining energy of UAV i and $E_{max}$ is the maximum energy capacity of a UAV. This normalization ensures that the energy values range between 0 and 1, where E=1 means the UAV has full energy and E=0 means the UAV has depleted its energy. Higher values of E indicate better energy efficiency, and CHs with higher energy are preferred because they can serve the cluster for a longer duration.

Our approach also checks the stability of the UAV mobility patterns and CH selection. The one with minimal changes in velocity and consistent patterns of movement is selected as the CH, thus ensuring that it may not move out of the cluster earlier. The UAV mobility pattern is defined as follows:

(8) $$MS\;\left(i\right)=\frac{1}{1+\sigma_{v}\left(i\right)},$$

where $\sigma_{v}(i)$ defines the standard deviation of velocity of UAV over the timeframe. A low oscillation in the velocity indicates more stability.

The distance D intra-cluster component considers the distance from the UAV to the other remaining UAVs that belong to its cluster. UAVs close to a cluster center are favored for the CH role because, consequently, communication costs at this point are reduced in all directions throughout the cluster. The formula to calculate D intra-cluster for UAV i is the average Euclidean distance between the UAV and every other UAV j in that same cluster as follows

(9) $$D\left(i\right)=\frac{1}{N_{cluster}}\sum_{j\in cluster}\sqrt{{\left(x_{i}-x_{j}\right)}^{2}+{\left(y_{i}-y_{j}\right)}^{2}+{\left(z_{i}-z_{j}\right)}^{2}},$$

where $N_{cluster}$ is the number of UAVs in the cluster. A lower value of D indicates that the UAV is closer to the CMs.

The number of one-hop neighbors is defined as the number of UAVs within the communication range $R_{max}$ of the UAV under evaluation. UAVs with more one-hop neighbors have a higher ability to facilitate communication within the cluster and are better placed to manage intra-cluster communication efficiently. The number of one-hop neighbors (NH) for UAV i is calculated as follows:

(10) $$NH\left(i\right)=\sum_{j=1}^{N}f\left(d_{ij}\right),$$

where NH(i) is the number of one-hop neighbors for UAV i, N is the total number of UAVs in the network, and $d_{ij}$ is the distance between UAV i and j. Note that $f\left(d_{ij}\right)=1$ if $d_{ij}<R_{max}$ (i.e., UAV j is within the communication range of UAV i) and $f\left(d_{ij}\right)=0$ otherwise.

UAVs with a higher number of one-hop neighbors are more likely to be selected as CH because they can effectively communicate with and manage more UAVs within their cluster. This metric is often combined with other factors (such as energy, mobility stability, and intra-cluster distance) in the fitness function for CH selection to ensure a well-balanced and efficient clustering process. The fitness function is defined as

(11) $$F_{CH}\left(i\right)=w_{1}\times E\left(i\right)+w_{2}\times MS\left(i\right)-w_{3}\times D\left(i\right)+w_{4}\times NH\left(i\right),$$

where value of $w_{1}$, $w_{2}$, $w_{3}$, and $w_{4}$ are 0.4, 0.3, 0.2, and 0.1, respectively. To prevent CH node failure, $w_{1}$ is considered to take residual energy as the highest priority. Following this, $w_{2}$ and $w_{3}$ are assigned second and third priority concerns to address UAV mobility patterns and minimize transmission costs between CMs and CHs, respectively. Finally, node degree $w_{4}$ (the highest number of one-hop neighbors) is considered the fourth priority for choosing the CH.

In this fitness function, a higher fitness score indicates a more suitable UAV for selection as the CH. UAVs with higher residual energy, greater mobility stability, closer proximity to CMs, and a higher number of one-hop neighbors achieve higher fitness values, making them more favorable candidates for CH selection. This prioritization ensures that the chosen CHs are energy-efficient, stable, centrally located, and well-connected, thereby promoting efficient and sustainable cluster management. HAMO-based clustering scheme is presented in Algorithm 1.
**Algorithm 1:** HAMO-based clustering.Input: Number of UAVs N$,\;3D\;\mathrm{positions}\;\mathrm{of}\;\mathrm{UAVs}\;P_{i}=(x_{i},y_{i},z_{i})$, velocity of UAVs $({Vx}_{i},{Vy}_{i},{Vz}_{i}$), UAV residual energy E(i)$,\;\mathrm{and}\;\mathrm{maximum}\;\mathrm{energy}\;\mathrm{level}\;\mathrm{of}\;\mathrm{UAVs}\;E_{max}$.$\mathrm{Output}\text{:}\;\mathrm{Initial}\;\mathrm{clusters}\;C_{k}$ with CHs# Step 1: Initialize population1: for each UAV i in {1, 2, …, N} do:2:    initialize UAV[i] with:$3\text{:}\;\;\;\;\;ID\text{:}\;{ID}_{i}$$,\;P_{i}=(x_{i},y_{i},z_{i})$, $({Vx}_{i},{Vy}_{i},{Vz}_{i}$), and E(i)4: end for# Step 2: Initial cluster assignment5: for each UAV i in {1, 2, …, N} do:$6\text{:}\;\;\;\mathrm{Calculate}\;\mathrm{distance}\;d_{ij}$, between UAV[i] and all other UAVs j using (1)7:   Assign UAV[i$]\;\mathrm{to}\;\mathrm{the}\;\mathrm{nearest}\;\mathrm{cluster}\;C_{k}$$\;\mathrm{based}\;\mathrm{on}\;d_{ij}$ 8: end for$\#\;\mathrm{Step}\;3\text{:}\;\mathrm{CH}\;\mathrm{selection}\;\mathrm{using}\;\mathrm{local}\;\mathrm{information}\;9\text{:}\;\mathrm{for}\;\mathrm{each}\;\mathrm{cluster}\;C_{k}\;\mathrm{do}\text{:}\;10\text{:}\;\;\;\mathrm{for}\;\mathrm{each}\;\mathrm{UAV}\;i\;\mathrm{in}\;C_{k}\;\mathrm{do}\text{:}\;11\text{:}\;\;\;\;\;\;\;\mathrm{Calculate}\;\mathrm{fitness}\;\mathrm{score}\;\mathrm{for}\;\mathrm{UAV}\;i\;\mathrm{based}\;\mathrm{on}\;(11)\;12\text{:}\;\;\;\;\;\;\;\mathrm{Select}\;\mathrm{the}\;\mathrm{UAV}\;\mathrm{with}\;\mathrm{maximum}\;\mathrm{fitness}\;\mathrm{score}\;F_{CH}\left(i\right)\;\mathrm{as}\;\mathrm{CH}\;13\text{:}\;\mathrm{end}\;\mathrm{for}\;\#\;\mathrm{Step}\;4\text{:}\;\mathrm{Output}\;\mathrm{summary}\;\mathrm{of}\;\mathrm{cluster}\;\mathrm{formation}\;14\text{:}\;\mathrm{Output}\;\mathrm{the}\;\mathrm{list}\;\mathrm{of}\;\mathrm{clusters}\;C_{k}\mathrm{with}\;\mathrm{their}\;\mathrm{corresponding}\;\mathrm{CHs}$

First, the proposed algorithm initializes the clusters and optimal selection of CHs. This approach uses local optimization to achieve efficient and stable clustering suitable for high mobility and dynamic conditions in FANETs. This aims to organize UAVs into clusters managed by energy-efficient and stable CHs, minimizing frequent re-clustering, and effective intra- and inter-cluster communication. Following initialization of the UAV population, an initial cluster assignment is performed. At the initial stage, every UAV calculates its Euclidean distance to join in a cluster based on this distance. This assignment only provides a rough structure in which clustering begins, thus forming the basis for optimized CH selection. The best UAVs as CHs in each cluster are determined using local information during the CH selection phase. For each UAV, there are four key metrics for computing a fitness score: energy level, mobility stability, intra-cluster distance, and number of one-hop neighbors. These four metrics are aggregated as a weighted sum to calculate the fitness score of each UAV in the cluster. Higher energy, stable mobility, proximity, and sufficient connectivity lead a UAV to a higher fitness score. Then, among all UAVs in the cluster, the one with the maximum fitness score is designated as the CH for that cluster. This selection approach optimally balances energy efficiency, stability, and connectivity for a dynamically energy-constrained FANET environment.

### 4.2. Cluster Maintenance Phase

Dynamical cluster maintenance is required to ensure network-wide efficient and balanced operation of UAVs in cases of high mobility. The clusters may also be overcrowded or skewed because UAVs move over time. This can cause performance degradation of the entire network. The goal of the second phase is to shift UAVs between their respective clusters in a manner that minimizes congestion, along with overall performance optimization and reduced-cost communications.

Each CH periodically calculates the local congestion factor (LCF) every t second to assess the level of congestion in its cluster. Congestion is determined based on the density of UAVs within the total area of the cluster. The LCF for cluster i is calculated as follows:

(12) $${LCF}_{i}=\frac{N_{cluster,\;i}}{A_{cluster,\;i}},$$

where $N_{cluster,\;i}$ is the number of UAVs in cluster i and $A_{cluster,\;i}$ is the area of cluster i. This formula directly measures the UAV density in a cluster. If ${LCF}_{i}$ is high, the cluster must be more congested because more UAVs are confined to a smaller area. Each CH compares its calculated ${LCF}_{i}$ with a given threshold value, representing the allowed maximum UAV density as follows:

(13) $${LCF}_{th}=\frac{Max\;acceptable\;UAVs}{Area}.$$

If the LCF of a cluster exceeds this threshold, it indicates that the cluster is congested and requires reformation. When the LCF of a cluster exceeds a threshold value, the CH initiates dynamic UAV reassignment to alleviate the congestion. This is done by finding neighbor clusters with lower LCF values and sufficient capacity to accommodate more UAVs. The CH of the congested cluster searches for neighboring clusters within a specified distance threshold. Only clusters within this distance are considered, ensuring minimal communication costs and energy consumption during reassignment. For each neighboring cluster j, the CH determines the number of additional UAVs that the cluster can handle without exceeding the LCF threshold. The maximum number of UAVs $N_{max,j}$ that a neighboring cluster j can accommodate is calculated as follows:

(14) $$N_{max,j}={LCF}_{th}\times A_{cluster,\;j}.$$

The available capacity $N_{ava}$ of neighboring cluster j is defined as

(15) $$N_{ava}=N_{max,j}-N_{current,\;j},$$

where $N_{current,\;j}$ is the current number of UAVs in cluster j and only clusters with $N_{ava}>0$ are eligible to receive the UAVs. The number of UAVs M to be moved from congested cluster i to neighboring cluster j is calculated based on the difference between the congested cluster’s LCF and the threshold as follows:

(16) $$M=\min\left(\alpha \times ({LCF}_{i}-{LCF}_{th})\times A_{cluster,\;i},N_{ava}\right),$$

where α is a scaling factor (typically 0<α≤10) to control reassignment rate, ${LCF}_{i}$ is the current LCF of congested cluster i, ${LCF}_{th}$ is the threshold LCF value that represents the maximum acceptable density for efficient communication, $A_{cluster,\;i}$ is the area of congested cluster i, and $N_{ava}$ is the available capacity of neighboring cluster j.

When the energy level of a CH drops below a predefined threshold $E_{th}$ ($E_{CH}<E_{th}$), the CH sends a control message to all its CMs, informing them about its low-energy status. This proactive notification ensures that the re-election process begins before the CH completely depletes its energy, thereby allowing for a smooth transition to a new CH. Once the notification is sent, the CMs participate in the voting process to elect a new CH.

Adaptive cluster maintenance refers to the process of dynamically adjusting and maintaining clusters of nodes within a network. Algorithm 2 aims to balance UAV density across clusters by dynamically reassigning UAVs when a cluster becomes congested. Each CH periodically calculates its LCF by dividing the number of UAVs by their cluster area. If the LCF exceeds a predefined threshold, indicating congestion, the CH searches for neighboring clusters within a specified distance that have lower LCF values and available capacities. For each eligible neighboring cluster, the CH calculates the number of UAVs that can be accepted based on the LCF threshold. Next, the CH selects the neighboring cluster, computes the quantity of UAVs to be transferred to that target using the difference in the LCF value at congested and the scaling factor, and proceeds to move them, updating their respective LCF values for both source and destination. This process ensures optimal UAV distribution, reduces congestion, and maintains an efficient network performance.
**Algorithm 2:** Adaptive cluster maintenance and congestion management.$\mathrm{Input}\text{:}\;\mathrm{List}\;\mathrm{of}\;\mathrm{clusters}\;\mathrm{with}\;\mathrm{information}\;\mathrm{on}\;\mathrm{the}\;\mathrm{number}\;\mathrm{of}\;\mathrm{UAVs}\;\mathrm{and}\;\mathrm{area}\;\mathrm{for}\;\mathrm{each}\;\mathrm{cluster},\;\mathrm{congestion}\;\mathrm{threshold}\;({LCF}_{th}$) for UAV density, distance threshold for identifying neighbouring clusters, and scaling factor for UAV reassignment.Output: Update distribution of UAVs between clusters and update LCF for each affected cluster.# For each time interval1: for each cluster i:2:     Calculate LCF using (12) # Check if LCF exceeds the threshold$3\text{:}\;\;\;\;\;\;\mathrm{if}\;{LCF}_{i}>{LCF}_{th}$ then # Identify neighboring clusters within the distance threshold4:       Eligible clusters = [ ]5:       for each neighboring cluster j within distance_threshold do6:          Calculate maximum and avaiable capacity of cluster j using (14) and (15) # Check the neighbor capacity and level of LCF$\begin{array}{l} 7\text{:}\;\;\;\;\;\;\;\;\;\;\;\;\mathrm{if}\;{LCF}_{j}>{LCF}_{th}\;\mathrm{and}\;N_{ava,j}>0\;\mathrm{then}\\ 8\text{:}\;\;\;\;\;\;\;\;\;\;\;\;\;\mathrm{Eligible}\;\mathrm{clusters}\;\mathrm{append}\;(j,\;N_{ava,j})\\ 9\text{:}\;\;\;\;\;\;\;\;\;\;\;\;\mathrm{end}\;\mathrm{if}\\ 10\text{:}\;\;\;\;\;\;\mathrm{end}\;\mathrm{for}\\ \#\;\mathrm{Select}\;\mathrm{the}\;\mathrm{neighboring}\;\mathrm{cluster}\;\mathrm{with}\;\mathrm{maximum}\;\mathrm{available}\;\mathrm{capacity}\\ 11\text{:}\;\;\;\;\;\;\mathrm{if}\;\mathrm{eligible}\;\mathrm{clusters}\;\mathrm{is}\;\mathrm{not}\;\mathrm{empty}\;\mathrm{then}\\ \#\;\mathrm{Calculate}\;\mathrm{number}\;\mathrm{of}\;\mathrm{UAVs}\;\mathrm{to}\;\mathrm{trasfer}\\ 12\text{:}\;\;\;\;\;\;\;\;\;\mathrm{Calculate}\;\mathrm{number}\;\mathrm{of}\;\mathrm{UAVs}\;\mathrm{to}\;\mathrm{transfer}\;\mathrm{using}\;(16)\\ \#\;\mathrm{Transfer}\;\mathrm{UAVs}\;\mathrm{from}\;\mathrm{congested}\;\mathrm{cluster}\;\mathrm{to}\;\mathrm{the}\;\mathrm{target}\;\mathrm{cluster}\\ 13\text{:}\;\;\;\;\;\;\;\;\;\mathrm{Transfer}\;\mathrm{UAVs}\;\mathrm{from}\;\mathrm{cluster}\;i\;\mathrm{to}\;\mathrm{target}\;\mathrm{cluster}\\ \#\;\mathrm{Update}\;\mathrm{LCF}\;\mathrm{for}\;\mathrm{both}\;\mathrm{clusters}\\ 14\text{:}\;\;\;\;\;\;\;\;\;\mathrm{Update}\;\mathrm{LCF}\;\mathrm{for}\;\mathrm{both}\;\mathrm{clusters}\;({LCF}_{i},\;{LCF}_{j}) \end{array}$ 15:       end if 16:    end if 17: end for

### 4.3. Routing Phase

Ferry nodes in the AO phase have become global explorers. They systematically collect network-wide data, including network density, congestion, and mobility patterns, to improve the CH selection and optimal positioning of nodes. These ferry nodes do not employ random levy flight exploration, but have a structured trajectory, crossing the network to collect detailed data from UAVs in their communication range. Levy flight, a type of random walk typically implemented for broad search capacity, is modified to leverage its search capability to combine short exploratory steps with long ones. The aggregation of such information by ferry nodes allows other UAVs to make good decisions regarding routing issues. Ferry nodes distribute real-time global data throughout the network, thereby providing UAVs with the information needed to optimize Cluster Head selection and adaptively change routing paths to improve network performance and resilience.

Algorithm 3 illustrates the function of the ferry nodes in a UAV network for gathering and diffusing global data. Given that ferry nodes move on a set path, they collect information from all UAVs within their communication range, including their position and load. This is comparable to levy flight exploration, in which ferry nodes methodically scan the network by gathering and summarizing essential metrics instead of depending on random motion. Once a UAV receives a control packet from its ferry node, it reports its updated status and the ferry node gathers this into a comprehensive global dataset of up-to-date network states. As the ferry nodes move along their path, they share the gathered information with neighboring UAVs, maintaining a fresh and dynamic view of the global network. Continuous packet exchange for control ensures that the network state is always updated, which aids in effective and adaptive decisions regarding clustering and routing within a FANET.
**Algorithm 3:** Ferry nodes for global information collection and distribution.Input: Predefined trajectory, UAV status (position and load), and control packets.Output: Collected global information (position and load from all UAVs) and distributed global information to neighbouring UAVs.# Initialize ferry nodes and begin traversal1: for each ferry node do:# Traverse network along the predefined trajectory 2:  while ferry node is operational:3:     Move ferry node along trajectory # Collect information from UAVs within communication range4:    for each UAV j within ferry node’s communication range do: # Request global information from UAV j5:         Send control packet (global_info) # UAV j responds with its status information6:         Receive info (Position j and load j) from UAV j # Aggregate received information7:         Store global info (Position j, load j) # Distribute collected global information to nearby UAVs 8:          for each UAV neighbor within communication range do:9:            Send global info (UAV neighbor and global info) # Perform levy flight exploration to cover the network area 10:             Execute levy flight (ferry node) 11:        end for 12:     end for 13:   end while 14: end for

HMAO uses both intra- and inter-cluster routing phases. Intra-cluster routing allows for efficient data aggregation because information is transmitted from UAVs in one cluster to the CH, which integrates information from its members. In inter-cluster routing, CHs are responsible for sending collected data from CMs to neighboring CHs. The energy-level constraints must be met before any UAV is considered for routing. Each UAV j must have a residual energy level greater than the $E_{th}$ to be eligible for selection in either the primary or the secondary path. If the residual energy is below this threshold, the UAV is excluded from the selection process. The residual energy of node $E_{j}$ is measured as follows:

(17) $$E_{j}=\frac{E_{remaing,j}}{E_{initial,j}},$$

where $E_{remaing,j}$ is a remaining energy of node j and $E_{initial,j}$ is the initial energy level of UAV node j.

After passing the energy checks, the UAV determines its fitness based on three critical parameters: the mobility, load, and congestion level. Predictive mobility-based routing aims to forward data towards the destination at high speed in a UAV that is moving toward the destination and at the same time has sufficient connectivity and is not moving in the opposite direction to the sender. This process enhances data delivery by selecting a suitable next-hop UAV based on predictions of future mobility, ensuring that the chosen UAV remains an efficient part of the communication chain. The future position of each neighboring UAV is predicted based on its current position and velocity. The predicted future position of UAV j after time interval ∆t is given by

(18) $$P_{j}^{future}=\left(x_{i},y_{i},z_{i}\right)+\left({Vx}_{i},{Vy}_{i},{Vz}_{i}\right)\times ∆t.$$

Using the predicted future position, the distance between the UAV’s future position and the destination is calculated as follows:

(19) $$d_{dest,\;j}^{future}=\left\|P_{j}^{future}-P_{dest}\right\|,$$

where $P_{j}^{future}$ is the UAV future position and $P_{dest}$ is the UAV destination. Moreover, the algorithm ensures that the UAVs move in the correct direction (toward the destination). For each UAV, the directionality and speed relative to the destination are computed as $\vec{V_{j}\cdot }\vec{V_{dest}}$, where $\vec{V_{j}}$ is the velocity of UAV j and $\vec{V_{dest}}$ is the velocity toward the destination. The effective range $R_{eff}$ is the predicted distance between the sender and forwarder UAV after ∆t. By ensuring that the selected forwarder node stays within the effective range most of the time, the algorithm prevents a situation in which the chosen forwarder moves out of range too quickly, disrupting the communication link. The mobility score for each neighboring UAV is computed by combining the directionality, proximity, remaining connectivity, and communication range stability, as follows:

(20) $${MS}_{j}=\left(\vec{V_{j}\cdot }\vec{V_{dest}}\right)+\lambda_{1}\left(\frac{1}{d_{dest,\;j}^{future}}\right)+\lambda_{2}\left(\frac{R_{eff}}{R_{max}}\right),$$

where the values of $\lambda_{1}$ and $\lambda_{2}$ are 0.6 and 0.4, respectively, $\frac{1}{d_{dest,j}^{future}}$ indicates the rewards for UAVs that will be closer to the destination in the future, and $\left(\frac{R_{eff}}{R_{max}}\right)$ measures the proportion of the communication range that will remain between the sender and forwarder nodes over time ∆t.

Load balancing ensures that data are distributed efficiently across UAVs, thereby preventing certain nodes from becoming overloaded. Load balancing in UAV networks is useful for delay reduction because it selects nodes with shorter queue lengths and higher packet processing rates to minimize the waiting time for packets and network latency. It allows the efficient utilization of resources because traffic spreads evenly across the network, thus preventing bottlenecks. Using this method helps prevent the overloading of certain UAVs, resulting in a more stable network that is not subject to node failures due to congestion. For each neighboring UAV j, load $L_{j}$ is calculated using both its queue length and packet rate, as follows:

(21) $$L_{j}=\beta_{1}\times \left({QL}_{j}\right)+{\;\beta }_{2}\left(\frac{1}{{PR}_{j}}\right),$$

where $\beta_{1}$ and $\beta_{2}$ are weighting factors that balance the importance of the queue length versus the packet rate and values of $\beta_{1}$ and $\beta_{2}$ are 0.6 and 0.4, respectively. ${QL}_{j}$ is the number of packets waiting in queue j of UAV node and ${PR}_{j}$ is the rate at which UAV j processes the packets (packets/s). The UAV with the lowest load is the most appropriate forwarding hop, as ${FH}_{j}=min\{L_{j}\}.$ If there is no congestion on the secondary path, the secondary path is turned off and the system selects the next-hop UAV based on the load on each UAV. In this scenario, the fitness function focuses on minimizing the loads on the selected nodes to determine efficient data routing. To determine the next-hop UAV j, the fitness value is calculated as follows:

(22) $${FV}_{j}=\min\left({(\delta }_{1}\times {MS}_{j})+\left(\delta_{2}\times {\;L}_{j}\right)\right),$$

where the values of $\delta_{1}$ and $\delta_{2}$ are 0.7 and 0.3, respectively.

Fault-tolerant secondary path routing for congestion avoidance is a strategy that improves the reliability and efficiency of packet delivery in FANETs. The system eradicates the priority-based mechanism and incorporates network conditions to dynamically tune routing such that packets bypass congested areas and reach their destination in time. The use of ferry nodes to monitor network congestion adds an adaptive element to the routing strategy, thus making it resilient against fluctuating network conditions, ensuring better load distribution, and minimizing the chances of packet loss or delays. The congestion level ${CL}_{net}$ is determined by combining the queue length, packet loss rates, and UAV density in a certain area as ${CL}_{net}=f(QL,\;PL,\;UD)$, where QL is the queue length, PL is the packet loss, and UD is the UAV density in a network. The value of the C threshold determines the degree of congestion to tolerate the primary path. As soon as congestion exceeds this value, it activates a redundant secondary path. The threshold value can be empirically derived from data or network conditions as ${CL}_{th}={(\varphi }_{1}max\;QL+\varphi_{2}\max PL+\varphi_{3}max\;UD)$, where values of $\varphi_{1}$, $\varphi_{2}$, and $\varphi_{3}$ are 0.5, 0.3, and 0.2, respectively. The proposed method activates the secondary routing path when congestion exceeds the threshold as follows:

(23) $$ARP=f\left(x\right)=\left\{\begin{array}{l} 1,\;\;\;\;if\;{CL}_{net}>{CL}_{th}\\ 0,\;\;\;\;otherwise \end{array}\right.,$$

where ARP represents the activate secondary path. If congestion is detected on the primary path, the UAV from the secondary list should be chosen based on its geographical position. The UAV provides a geographically unique direction from the path taken by the primary node, measured by the angle between the paths as follows:

(24) $$A_{s}>A_{th}=\cos^{-1}\left(\frac{V_{pri}\cdot V_{sec}}{\left|V_{pri}\right|\left|V_{sec}\right|}\right),$$

where $A_{s}$ and $A_{th}$ represent the angle of secondary path and angle of threshold, respectively. The threshold angle indicates the minimum angle between the primary and secondary paths. $V_{pri}$ and $V_{sec}$ are the direction vectors of the primary and secondary paths.

The final fitness value used to select the next-hop UAV for either the primary or secondary path considers both the mobility prediction and congestion control. The fitness function is defined as

(25) $${FF}_{j}={(\delta }_{1}\times {MS}_{j})+\left(\delta_{2}\times L_{j}\right)+\left(\delta_{2}\times \frac{1}{{CL}_{j}}\right),$$

where the values of $\delta_{1}$, $\delta_{2}$, and $\delta_{3}$ are 0.5, 0.3, and 0.2, respectively.

Algorithm 4 implements a congestion-aware fault-tolerant routing mechanism for UAV networks that incorporates energy constraints, mobility prediction, load balancing, and congestion control. Initially, each UAV gathers position, velocity, energy, queue length, and packet rate data from its neighbors through periodic Hello Messages. In the eligibility phase, it filters out only those UAVs that have residual energy higher than a certain threshold value, and discards low-energy UAVs well in advance to prevent premature network depletion. During the fitness evaluation phase, eligible UAVs are scored for the mobility and load balancing criteria. Mobility is scored with a view of predicted future position, distance towards the destination, and directionality of motion, giving preference to the UAV moving toward the destination. The load is determined by the queue length and packet processing rate, and the combined fitness score of the mobility and load is used to select the optimal next-hop UAV. Congestion monitoring is conducted by ferry nodes, which assess network density, queue lengths, and packet loss rates. The second path is dynamically activated. It selects nodes that meet geographical separation, load, and energy requirements such that interference with the primary path is avoided when congestion exceeds a certain threshold. Data packets are forwarded through primary or both paths according to real-time network conditions to ensure efficient load distribution and high fault tolerance. Our proposed approach is inspired by the principles of AO, where ferry nodes act as global information hubs, sharing network-wide congestion and load conditions with the UAVs. This allows UAVs to dynamically adjust routing decisions based on a real-time overview of network conditions, thereby enhancing their robustness and adaptability in high-mobility environments.
**Algorithm 4:** Congestion-aware fault-tolerant routing.$\mathrm{Input}\text{:}\;\mathrm{Positions}\;\mathrm{of}\;\mathrm{UAVs}\;P_{i}=(x_{i},y_{i},z_{i})$, velocity of UAVs $({Vx}_{i},{Vy}_{i},{Vz}_{i}$), residual energy level of UAVs E(i), congestion data from ferry nodes, queue length QL, packet rate PR, and neighbor table NT.Output: Primary path and secondary path.# Step 1: Initialize UAV parameters1: for each UAV i  do:$2\text{:}\;\;\;P_{i}=(x_{i},y_{i},z_{i})$, $V_{i}=({Vx}_{i},{Vy}_{i},{Vz}_{i})$, $E\left(i\right)=E_{i}$$,\;{QL}_{i}=0$$,\;{PR}_{i}=0$$,\;\mathrm{and}\;{NT}_{i}=\{\;\}$3: end for # Step 2: Periodically send Hello Messages and collect neighbor data4: for each UAV i  do:5:   Broadcast (Hello Message)6: end for 7: for each UAV i  do:8:   Receive Hello Message from neighbor j $9\text{:}\;\;\;\mathrm{Update}\;{NT}_{i}=\{P_{j},\;V_{j},\;E_{j},\;{QL}_{j},\;{PR}_{j}\}$ 10: end for# Step 3: Ferry nodes collect global information (density and congestion)11: for each ferry node do:12:   Collect positions of all UAVs within communication range $13\text{:}\;\;\;\mathrm{Calculate}\;\mathrm{the}\;regional\;density=\frac{Number\;of\;UAV\;in\;Area}{Area\;size}$ 14:   Share the density and congetion level with all UAVs15: end for # Step 4: Eligibility phase based on energy threshold 16: for each UAV i do: 17:   Eligible nodes=[ ]18:   for each neighbor j of UAV i do: # Check if neighbor j has sufficient residual energy $19\text{:}\;\;\;\;\;\;\mathrm{if}\;E_{j}\geq E_{th\;\mathrm{then}}$ 20:       Eligible nodes append (j) // Add j to list of eligible nodes 21:      end if 22:    end for 23: end for # Step 5: Fitness evaluation for eligible nodes (primary path) 24: for each UAV i  do: 25:    Best fitness value BFV=−1 26:    Selected forwarder=None 27:    for each eligible neighbor j in eligible nodes do # Predict future position of UAV j based on mobility 28:       Predict the future position using (18) # Calculate distance from future position to destination 29:       Calculate the distance from future position to destination using (19) # Calculate directionality score (if moving toward destination) $30\text{:}\;\;\;\;\;\;\;\mathrm{Calculate}\;\mathrm{the}\;\mathrm{direction}\;{Dir}_{j}=(V_{j},(destination-P_{j})$ $31\text{:}\;\;\;\;\;\;\;\;\;\;\mathrm{if}\;{Dir}_{j}>0$ then 32:         Calculate the mobility score and load for neighbor j using (20) and (21) 33:         Calculate the fitness value using (22) $34\text{:}\;\;\;\;\;\;\;\;\;\;\;\;\;\mathrm{if}\;{FV}_{j}>BFV$ then 35                $BFV={FV}_{j}$ and select forwarder node j 36:              end if 37:          end if 38:    end for 39: end for # Step 6: Congestion monitoring by ferry nodes for secondary path activation 40: for each ferry node do: 41:   Collect QL, PL, UD$\;\mathrm{and}\;\mathrm{measure}\;\mathrm{the}\;\mathrm{congestion}\;\mathrm{level}\;\mathrm{by}\;{CL}_{net}=f(QL,\;PL,\;UD)$ 42:     for each UAV i in region do 43:       Send CL to UAV 44:      end for 45: end for # Step 7: Decision logic for fault-tolerant secondary path activation 46: for each UAV i  do: $47\text{:}\;\;\;\;\mathrm{if}\;{CL}_{net}>{CL}_{th}$ then 48:         Activate ARP=True 49:    else Activate ARP=False 50:    end if 51: end for # Step 8: Secondary path selection for congestion avoidance (if activated) 52: if activate ARP then 53:    for each eligible neighbour k of UAV i not on the primary path do 54:         Ensure geographic separation from primary path using (24) $55\text{:}\;\;\;\;\;\;\;\;\;\;\;\mathrm{if}\;\mathrm{direction}\;\mathrm{seperation}\;k>A_{th}$ then 56:             Calculate the load for secondary path candidate k 57:             Calculate the fitness function for secondary path candidate k using (25) $58\text{:}\;\;\;\;\;\;\;\;\;\;\;\;\;\;\;\;\mathrm{if}\;{FF}_{k}>BFV$ then 59:                   $BFV={FF}_{k}$ 60:                 end if 61:           end if 62:    end for 63: end if # Step 9: Forward data via selected paths 64: for each UAV i do: 65:   if activate secondary path ARP then 66:    Forward packet (selected forwarder)   // Primary Path 67:    Forward packet (selected secondary forwarder) // Secondary Path 68:   else forward packet (selected forwarder)   // Only Primary Path 69:   end if 70: end for

**Figure 3:**
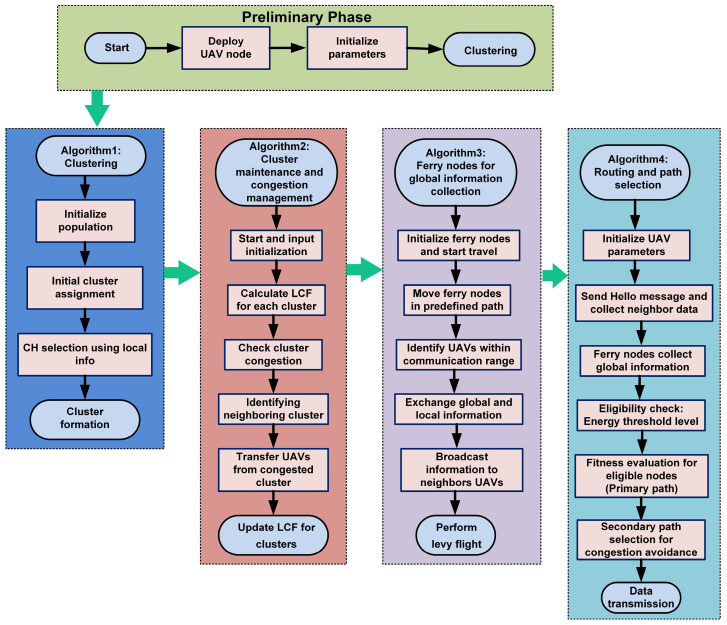
Algorithm flow of proposed HMAO scheme.

Figure 3 illustrates the flowchart of the proposed algorithm. It contains four stages: clustering, cluster maintenance and congestion management, ferry node global information collection and distribution, and routing and path selection (primary and secondary).

### 4.4. Computational Complexity

The computational or time complexity of proposed HMAO is represented as $O(W\;*\;d\;*\;t_{max})$, where W is the size of the population, d the dimension of the optimization problem, and $t_{max}$ the maximum number of iterations. In the initialization phase, the algorithm requires O(W ∗ d) time to calculate the control parameters. After that, the algorithm requires O(W ∗ d) to update three best solutions in each iteration. For the maximum number of iterations, the total time complexity is $O(W\;*\;d\;*\;t_{max})$.

## 5. Performance Evaluation

In this section, the performance of the proposed HMAO clustering and routing protocol is analyzed in comparison with other routing protocols such as MFO-based clustering [9], MWCRSF [27], DCFH [28], DCM [31], and HMGOC (MGO-JAYA) [33]. This analysis is performed by using Network Simulator 3 (NS-3.35), which is an event-driven wireless network simulator that is well suited for simulating UAV networks because of its ability to accurately process events and model a wide variety of networks. In this study, we compared our proposed HMAO with five other existing schemes in terms of cluster stability, PDR, overhead, delay, and energy consumption. Detailed definitions of these performance metrics can be found in [17,24].

### 5.1. Simulation Environment

The simulations were conducted on NS-3.35, operating on a system equipped with an Intel Core i7 processor and 16 GB RAM. The simulated network environment comprised six distinct scenarios with varying UAV configurations that are capable of ad hoc communication within a 3D box-shaped area. The UAVs operated under a Gauss–Markov mobility model to account for realistic dynamic movement patterns. Each UAV node was equipped with an IEEE 802.11ac wireless network interface for ad hoc communication, operating at a transmission power of 20 dBm. The simulation time was set to 120 s, and identical simulation parameters were maintained across all the routing protocols. The simulation parameters are listed in Table 2.

### 5.2. Results and Discussion

The proposed HMAO algorithm was compared across key performance metrics, including CH count, CH changing ratio, PDR, routing overhead, latency, and energy consumption, to demonstrate its superior performance in FANETs.

**Figure 4:**
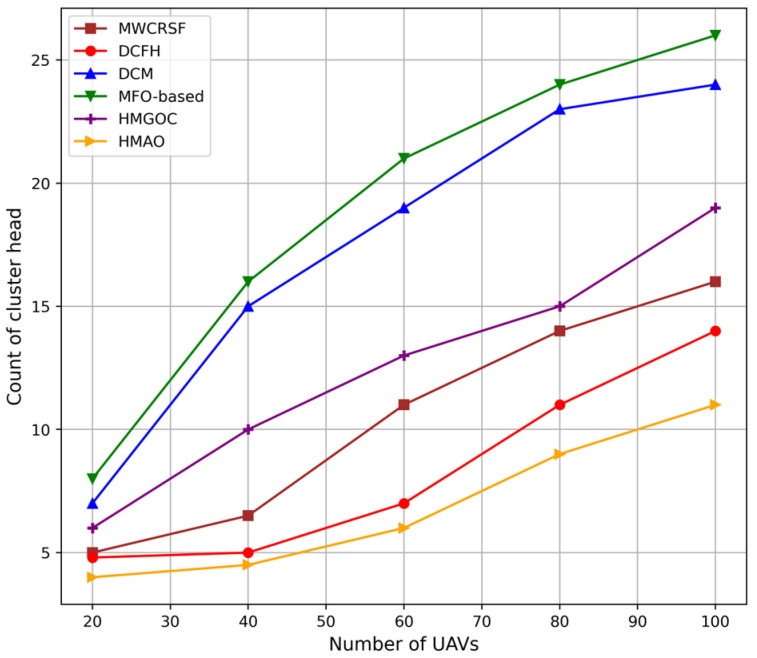
Count of CHs vs. number of UAVs.

Figure 4 depicts how the HMAO algorithm maintains an optimal number of CHs as the number of UAVs increases, which is more than that of other algorithms that tend to create extra clusters, thus leading to inefficient energy consumption and instability. The proposed HMAO algorithm significantly enhances the clustering efficiency by selectively forming CHs based on adaptive criteria designed according to the positioning and dynamic conditions of UAVs. This technique reduces redundant CHs, saves energy, and provides stable network connectivity, showing significant improvements in high-mobility environments. Other algorithms, such as MFO, DCM, and MWCRSF, cannot prevent excessive clustering caused by strict clustering conditions. MFO lacks dynamic adaptability, thus causing frequent and energy-hungry re-clustering. DCM and MWCRSF are weak at load balancing and optimal CH selection. Shifting network demands result in decreased network stability and higher energy consumption.

**Figure 5:**
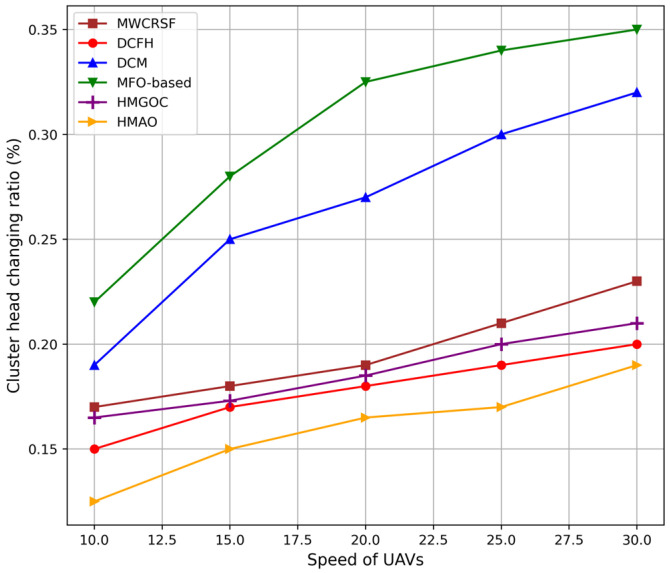
Comparison of CH changing ratios.

Figure 5 shows that the low CH change ratio in the proposed HMAO is due to its adaptive and stable clustering mechanism, which reduces the frequent re-election of cluster heads. In HMAO, this selection process of CHs depends on long-term factors, such as UAV stability, energy levels, and connectivity. In addition, HMAO adapts dynamically to minor changes without constantly reconfiguring the clusters. This maintains a stable CH structure and reduces the overhead from the frequent CH switches.

**Figure 6:**
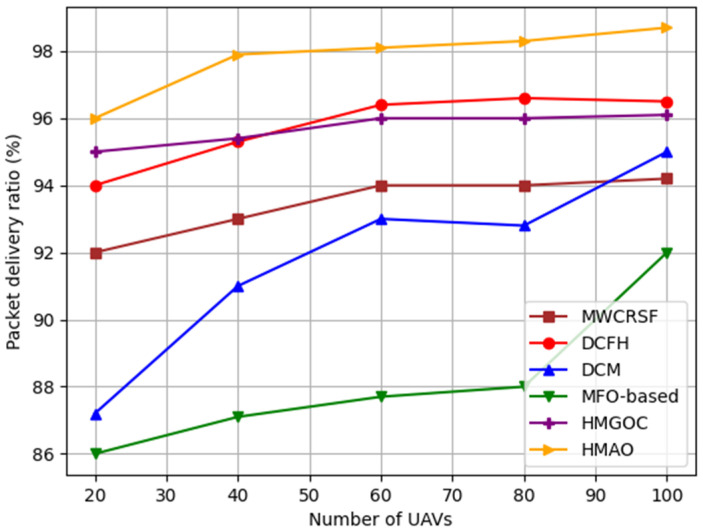
PDR vs. number of UAVs.

HMAO achieves a high PDR as its predictive routing mechanism, using global information through the AO, ensures stable paths, and minimizes packet loss even with an increasing number of UAVs. HMGOC focuses on selecting stable CHs using MGO but lacks the adaptive, global perspective provided by the AO in HMAO. This limitation leads to a lower PDR in dynamic scenarios where rapid UAV movement results in frequent link changes. MWCRSF uses weighted metrics for clustering based on mobility, but it does not adapt routes dynamically to global conditions. Consequently, it experiences higher packet loss when the UAV density increases because of its inability to predict movements and maintain stable paths. DCM adjusts clusters based on local metrics but fails to adapt routing decisions to network-wide changes, leading to a decrease in PDR as the number of UAVs increases, owing to inefficient handling of mobility. MFO focuses on optimizing clustering through moth-inspired search patterns; however, it lacks the routing efficiency and global adaptability of HMAO, leading to increased packet loss in highly mobile conditions where stable routing is crucial. DCFH improves clustering with the fire hawk optimizer, but its routing mechanisms are reactive, not predictive. Thus, DCFH suffers from packet loss with high UAV mobility, for which proactive path selection is required. Figure 6 shows that with an increased number of UAVs, the HMAO algorithm proposed here offers a higher PDR than the other methods. This is because of its stable clustering and routing, which reduce packet loss in dynamic environments.

**Figure 7:**
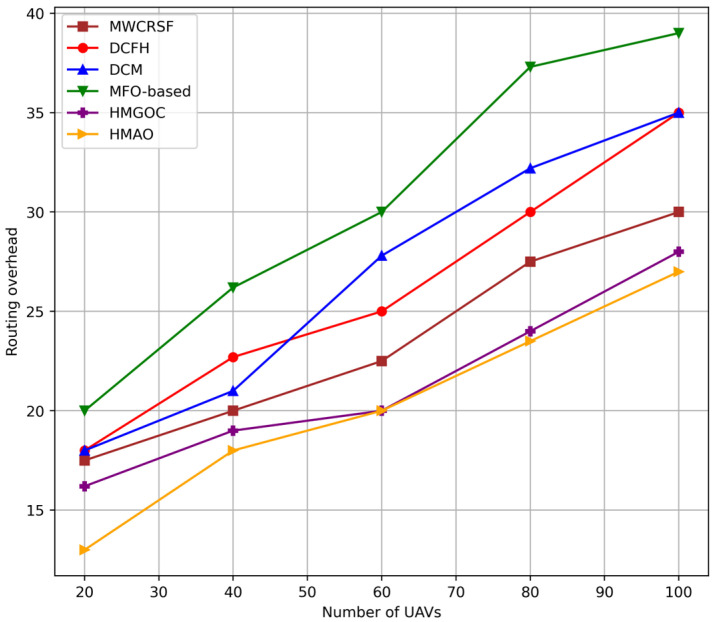
Routing overhead vs. number of UAVs.

With an increased number of UAVs, HMAO has low routing overhead, as depicted in Figure 7. This is because once stable clusters are merged by using MGO with adaptive routing having AO, there arises a low-frequency update of routes as compared with a regular communication process with routing overhead. The local information of HMAO can be used for the fine-tuning of clustering, which may reduce the frequency update, resulting in a need for no re-clustering. Although HMGOC results in efficient clustering, the scheme still suffers from more significant routing overhead because there is no global optimization layer, such as AO. AO can use global information and perform congestion-aware routing, whereas HMGOC is less capable of predicting UAV density and mobility changes, leading to more route adjustments and, in turn, more overhead. MWCRSF uses mobility metrics for better initial cluster formation, but it lacks the global awareness that HMAO has in optimizing routes based on network-wide conditions. This leads to increased overhead because UAV movements cause repeated rerouting efforts. DCM dynamically adjusts clusters, but fails to manage overhead when the number of UAVs increases. It lacks a mechanism for prediction and reacts only after changes occur, thereby increasing the control message exchange and overhead. MFO performs well in initial clustering but has a relatively higher overhead in routing compared to HMAO because it does not utilize global data for proactive adjustments, and hence, more frequent recalculations of the route. DCFH adapts to clustering based on local conditions but does not account for a global view in routing. Therefore, it brings about more overhead because it has to update routes frequently owing to difficulty adapting and coping with alterations in different network densities and UAV movement patterns accordingly.

**Figure 8:**
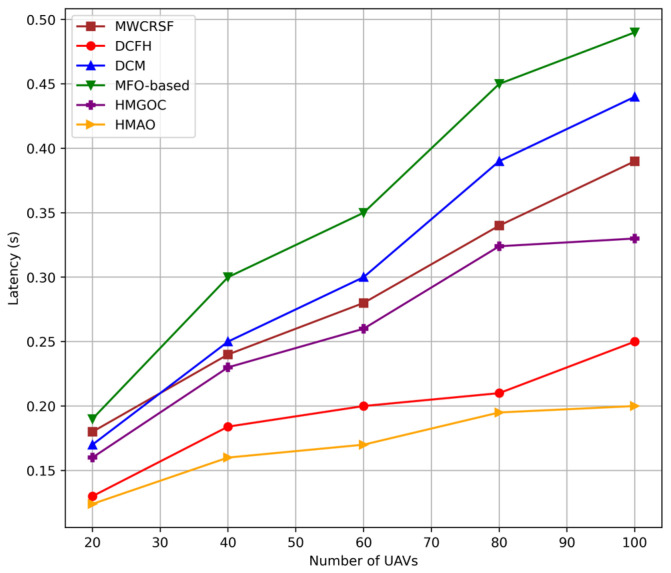
Latency (s) vs. number of UAVs.

Figure 8 indicates that, with a higher number of UAVs, the latency is less than that of HMAO. The reason for this phenomenon is that the AO predicts its routes and makes one that is not congested, thereby resulting in less delay. HMAO reaches a low latency because of the predictive capability of AO, which opts for routes that, based on changing numbers of UAVs over time, would not be different. Thus, with an increase in the number of UAVs in a network, data delivery also occurs quickly because it adjusts routing in proportion to the changes predicted by UAVs. HMGOC is good at selecting a stable head of the cluster, but is also a high-latency scheme owing to its reactive routing behavior. In the absence of predictive routing mechanisms, such as AO, it lags in adapting to changes in the network. The reduced latency initially owing to the use of mobility-based clustering makes MWCRSF weak in high-mobility environments where the routing path keeps changing. It suffers from a lack of a global perspective while routing, and thus delays adjustments and increases the latency. As the network size increases, the local updates of DCM for clustering create higher latency because it takes time to update the cluster structures and routes after the movement of the UAVs. The routing mechanisms of MFO are efficient but do not investigate global mobility patterns.

**Figure 9:**
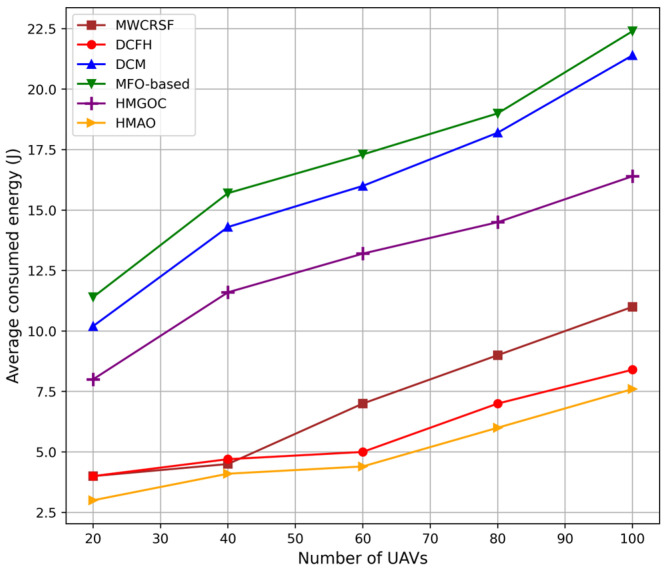
Average energy consumption (J) vs. number of UAVs.

As shown in Figure 9, HMAO consumes less energy than HMGOC, MWCRSF, DCM, MFO, and DCFH as its approach performs dual optimization for local and global optimization. Therefore, in HMAO, MGO selects energy-efficient, stable CHs based on the residual energy and mobility stability that reduce events of frequent re-clustering. At the same time, in AO, global information is used to route the packet, minimizing link failures and low re-routing. This saves the energy costs of transmission. Furthermore, load balancing and congestion-aware secondary path activation of HMAO reduce energy-intensive node overloading and offer smooth data delivery, such that energy is conserved compared to other methods that do not integrate these optimum mechanisms.

Other algorithms, such as HMGOC, MWCRSF, DCM, MFO, and DCFH, are characterized by very high energy consumption, mainly because of the occurrence of repeated re-clustering and inefficient routing. In the HMGOC and MWCRSF algorithms, predictive routing and load balancing are not included, indicating that the stability of clusters is unstable and consumes considerable energy through repeated re-clustering processes. In the cases of DCM and DCFH, although dynamic clusters are supported, failure to utilize global information about the network results in frequent link breaks and re-routing, which leads to energy consumption. MFO has a simpler optimization strategy with larger data paths associated with higher transmission costs, whereas HMAO possesses attributes such as adaptive clustering and fault tolerance.

## 6. Conclusions

The high mobility and fast-changing nature of UAVs bring several challenges to clustering and routing in FANETs. In this study, we proposed a hybrid bio-inspired algorithm, HMAO, which combines the strengths of MGO and AO to enhance clustering stability and data delivery reliability. Leveraging global information, HMAO integrates MGO for efficient CH selection and AO for routing, achieving load balancing and fault-tolerant path selection. Through local optimization, MGO maintains stable CHs by considering UAV energy levels, mobility patterns, intra-cluster distance, and one-hop neighbors, reducing re-clustering frequency, and ensuring efficient cluster coordination. For clustering maintenance, HMAO employs a congestion-based approach that redistributes UAVs in overloaded clusters, addressing communication imbalances. Its predictive routing mechanism uses mobility data to establish reliable and adaptive multi-hop paths, minimizing packet loss in high-mobility scenarios. Additionally, fault-tolerant secondary path activation optimizes energy consumption while ensuring resilient data transfer. HMAO’s density-aware clustering further enhances adaptability, enabling efficient UAV resource management under varying network loads. Simulation results demonstrate that HMAO outperforms existing protocols in terms of PDR, delay, overhead, and energy consumption, making it a scalable and high-performance solution for dynamic FANETs.

Despite its proven advantages, HMAO has some limitations that create avenues for further research. A primary drawback of HMAO is that it relies heavily on real-time network condition assessments, which can be problematic in highly congested networks and resource-constrained environments. Further improvements could also be made to how HMAO adapts to extreme mobility patterns, such as swarm-based UAV formations, for better response times under dynamic network topology changes. These may include further research studies on enhancing the performance of HMAO in predicting UAV movements. By addressing these limitations and exploring novel extensions, future research can contribute to more refined and adaptable FANET solutions that balance the computational demands with network performance. Furthermore, we may test the HMAO algorithm under practical conditions to verify its convergence and robustness.

## Figures and Tables

**Table 1:** Qualitative comparison of existing clustering and routing techniques for FANETs.

Reference	Approach	Objective	Strengths	Weaknesses	Scalability and Adaptability
EARVRT [26]	Energy-aware routing with virtual routing tables	Reducing unnecessary control messages	Reduced routing overhead, high adaptability, improved packet delivery rate (PDR), and reduced latency	Limited network scalability	Moderate scalability and performs well in moderate mobility scenarios
LFEAR [27]	Local filtering enhanced AODV routing	Enhancing AODV with local filtering	Enhanced adaptability, managed routing overhead, and improved route stability	Higher routing overhead compared to EARVRT	Adaptable in moderate mobility and limited by overhead in high-density FANETs
K-MORP [28]	K-means based mobility-aware routing	Efficient routing through clustering	Reduces routing overhead, enhances scalability, and resource management	Complexity in cluster formation and stability issues at high speeds	High scalability and effective in controlled environments
MWCRSF [29]	Mobility-based weighted clustering scheme	Enhancing FANET performance through optimized routing	Improves load balancing and communication efficiency	High computational complexity for real-time data processing	Good scalability but limited adaptability in dynamic environments
DCFH [30]	Dynamic clustering for fault handling	Improving fault tolerance in dynamic FANETs	Efficient in managing faults and improving data reliability	Limited scalability due to complexity	Moderate scalability and suitable for low to moderate mobility scenarios
ICW [31]	Centralized clustering scheme based on WOA	Enhancing energy efficiency and stability	Low clustering time, achieves high PDR and low delay	Computational high overhead due to centralized clustering process	Adaptable to dynamic FANET and address high-speed UAV mobility and link stability
UF-GPSR [32]	Modified geographic perimeter stateless routing	Stable and efficient data dissemination	High data delivery ratio, efficient throughput, and reduced latency	Limited scalability and lacks adaptive hello message interval adjustment	Moderate scalability and effective in structured UAV deployments
DCM [33]	Dynamic clustering mechanism	Enhancing efficiency in communication and resource allocation	Improves reliability and operational efficiency	High computational complexity in dynamic scenarios	Moderate to high scalability and adaptable in moderate mobility FANETs
SOCS [24]	Self-organization-based clustering scheme	Enhancing network management and reliable communication	Increases communication reliability and network stability	High computational requirements	High scalability in structured deployments
MFO [11]	MFO-based routing	Enhancing energy efficiency and path stability	High convergence speed and energy-efficient path selection	Performance degradation in high-density environments	Moderate adaptability and suited for static to moderate mobility FANETs
HMGOC MGO-JAYA [35]	Hybrid MGO-JAYA for cluster optimization	Achieving energy-efficient CH selection	Balances energy and cluster stability	Limited adaptability to global conditions	Moderate scalability
BICSF [36]	Mobility-based weighted clustering scheme	Enhancing FANET performance through optimized routing	Improves load balancing and communication efficiency	High computational complexity for real-time data processing	Good scalability but limited adaptability in dynamic environments

**Table 2:** Simulation parameters.

Parameter	Value
Network size	2000 m × 2000 m × 500 m
Simulation time	120 s
UAV speed	10–30 m/s
Number of UAVs	20–100
UAV communication range	300 m
Minimal separation between UAVs	10 m
Mobility model	Gauss–Markov mobility model
Air density	1.23 kg/m^3^
Channel model	IEEE 802.11n
Inter-cluster carrier frequency	5 GHz
Intra-cluster carrier frequency	2.4 GHz
Acceleration due to gravity	9.8 m/s^2^
Packet size	512 bytes
Packet generation model	Constant bit rate (CBR), 2 Mbps
Initial energy per UAV	2000 J
Energy consumption model	Communication energy
Electronic energy per Bit	50 nJ/bit
Amplification energy (Long range)	0.01 pJ/bit/m^4^
Amplification energy (Short range)	100 pJ/bit/m^2^

## Data Availability

Data will be made available on request.
